# Neuronal calcium sensor proteins are unable to modulate NFAT activation in mammalian cells

**DOI:** 10.1016/j.bbagen.2007.10.011

**Published:** 2008-02

**Authors:** Daniel J. Fitzgerald, Robert D. Burgoyne, Lee P. Haynes

**Affiliations:** The Physiological Laboratory, School of Biomedical Sciences, University of Liverpool, Crown Street, Liverpool, L69 3BX, UK

**Keywords:** Ca^2+^, calcium, NFAT, Nuclear Factor of Activated T-cells, NCS, Neuronal Calcium Sensor, CaBP1, Calcium Binding Protein-1, CaM, Calmodulin, CN, Calcineurin, GST, Glutathione-*S*-transferase, KChIP1, Potassium Channel Interacting Protein-1, GFP, Green Fluorescent Protein, SDS, Sodium Dodecyl Sulphate, PAGE, Polyacrylamide Gel Electrophoresis, HRP, Horse Radish Peroxidase, HEPES, 4-(2-Hydroxyethyl)piperazine-1-ethanesulfonic acid, EDTA, Ethylenediaminetetraacetic acid, EGTA, Ethylene glycol-bis(2-aminoethylether)-*N,N,N′N′*-tetra-acetic acid, NTA, *N,N*-Bis(carboxymethyl)glycine, PMA, Phorbol 12-myristate 13-acetate, NCS family proteins, Calmodulin, Calcineurin activity, NFAT signaling

## Abstract

Calcium activated gene transcription through *N*uclear *F*actor of *A*ctivated *T*-cells, (NFAT) proteins, is emerging as a ubiquitous mechanism for the control of important physiological processes. Of the five mammalian NFAT isoforms, transcriptional activities of NFATs 1-4 are stimulated by a calcium driven association between the ubiquitous phosphatase calcineurin and the calcium-sensing protein calmodulin. Published in vitro evidence has suggested that other members of the calmodulin super-family, in particular the neuronal calcium sensor (NCS) proteins, can similarly modulate calcineurin activity. In this study we have assessed the ability of NCS proteins to interact directly with calcineurin in vitro and report a specific if weak association between various NCS proteins and the phosphatase. In an extension to these analyses we have also examined the effects of over-expression of NCS-1 or NCS-1 mutants on calcineurin signalling in HeLa cells in experiments examining the dephosphorylation of an NFAT-GFP reporter construct as a readout of calcineurin activity. Results from these experiments indicate that NCS-1 was not able to detectably modulate calcineurin/NFAT signalling in a live mammalian cell system, findings that are consistent with the idea that calmodulin and not NCS-1 or other NCS family proteins is the physiologically relevant modulator of calcineurin activity.

## 1 Introduction

Calcineurin (CN) [1] is a serine/threonine specific protein phosphatase present in all eukaryotes that is responsible for the modulation of numerous cell signalling pathways. Calcineurin interacts with the ubiquitous EF-hand containing calcium (Ca^2+^) sensor calmodulin (CaM) [2] and this obligate association drives calcium dependent phosphatase activity, one of the primary cellular targets of which are the *N*uclear *F*actor of *A*ctivated *T*-cell (NFAT) family of transcription factors. This family of gene regulatory proteins is composed of five members (NFAT1-5) [3,4], that were first identified as fundamental components of the recombinatorial immune system of vertebrates [5], but which have now been implicated in a far broader range of diverse and important physiological processes. Expression of NFAT proteins is not restricted to cells of the immune system, demonstrated by their presence and effect on key functions in a selection of other cell and tissue types including brain and muscle. In a neuronal setting, NFATs have been implicated in axonal remodelling, synaptic plasticity and memory function [6-9]. Transcriptional events mediated by activated NFATs have been linked to aspects of cardiovascular development [10,11] and they have also been suggested to drive proliferative and apoptotic pathways in epithelial cells, fibroblasts, pre-adipocytes, pancreatic β-cells and osteoblasts [12-16]. The discovery that NFAT proteins can influence cell cycle regulation has led to the proposal that they may also be involved in certain aspects of tumorigenesis [17].

NFAT proteins in unstimulated cells are heavily phosphorylated on multiple serine residues by of a number of constitutively active kinases [18]. This modification acts to mask a nuclear localisation signal and under such circumstances NFATs remain predominantly cytosolic and transcriptionally inactive. Stimulation of cells with agonists coupled to the phospholipase-C/IP_3_ generating pathway, depletion of endoplasmic reticulum Ca^2+^ stores and subsequent sustained elevation of intracellular Ca^2+^ concentration ([Ca^2+^]_i_) due to opening of plasma membrane Ca^2+^ release activated Ca^2+^ channels elicits the rapid dephosphorylation of NFAT proteins, their nuclear import and upregulation of target gene expression [3]. Under such circumstances, the Ca^2+^ signal is integrated upstream of NFAT by CN in conjunction with CaM and it is this protein complex that represents the critical regulatory mechanism for NFAT dephosphorylation and hence activation of NFAT dependent gene expression. The relationship between CN/CaM and NFATs is so deeply intertwined that nuclear translocation of NFAT proteins has been employed as a robust assay of CN activity in intact mammalian cells [19-21] and has even found application in large scale screens for proteins involved in calcium induced calcium release [22].

CaM is the primordial member of a superfamily of small EF-hand containing Ca^2+^ binding proteins that function to transduce spatial and temporal cellular Ca^2+^ signals. Other members of the CaM family include the neuronal calcium sensor (NCS) subgroup of neuronal/neuroendocrine specific proteins [23,24]. Although only distantly related (21% sequence identity between CaM and NCS-1) published in vitro studies have identified potential overlap in target protein interactions between CaM and NCS-1 including an apparent positive interaction of both proteins with CN [25]. Since CaM exhibits high and ubiquitous expression these observations led to the speculation that NCS proteins may simply represent redundant or alternative modulators of classical CaM targets. Evidence from our laboratory and others has gone some way to disproving this idea with the identification of NCS specific binding proteins that, at present, have no documented interaction with CaM [26]. Further evidence for distinct functionality of the NCS protein family is apparent from their higher Ca^2+^ affinity compared to CaM, their differential cellular/tissue distributions and their non-redundancy revealed in genetic studies [23,27]. Together these data indicate that NCS proteins possess the requisite biochemical properties to allow them respond to unique Ca^2+^ signals and to bind to distinct effector proteins. The possibility exists however that overlap between CaM and NCS target specificities may be of physiological significance and in the case of CN, identification of a functionally relevant interaction with NCS proteins would be of great interest in view of recent findings implicating CN activity in aspects of neuronal function.

In order to resolve some of the outstanding questions concerning potentially redundant functions of NCS proteins we have examined whether or not they are able to elicit CN activation in the physiologically relevant setting of intact mammalian cells using NFAT activation as a coupled reporter. We present in vitro biochemical data indicating that various recombinantly expressed NCS family members are able to directly bind CN although to lower levels compared with CaM. Consistent with these data we have also determined that expression of NCS-1, mutants of this protein that have previously been demonstrated to disrupt NCS-1 function in vivo, along with other NCS family members have no effect on NFAT dephosphorylation in live HeLa cells in response to elevation of [Ca^2+^]_i_. These data are supported by studies indicating that the nuclear translocation of NFAT on elevation of [Ca^2+^]_i_ to cell nuclei is unaffected in cells expressing NCS-1 or NCS-1 mutants. We therefore suggest that NCS proteins, although exhibiting a detectable in vitro affinity for CN, may not be likely to act as physiologically relevant modulators of CN activity or NFAT mediated gene transcription in intact cells.

## 2 Materials and methods

### 2.1 Recombinant proteins

All recombinant GST fusion proteins used in this study were expressed and purified as previously described [26]. Purified recombinant calcineurin was obtained from Sigma (Poole, UK) or Biomol (Exeter, UK). Unless otherwise stated all chemicals were of analytical grade and obtained from Sigma.

### 2.2 Small Scale GST protein binding assays

In all binding assays, recombinant GST-fusions proteins (1 μM) were immobilised by incubation with 40 μl of glutathione cellulose (GST cellulose) resin (50% bead slurry, Bioline, London, UK) that had been pre-washed in binding buffer (KCl 50 mM, HEPES 20 mM (pH 7.4), EGTA 5 mM, NTA 5 mM, CaCl_2_ 4.6 mM (giving a free [Ca^2+^] of 1 μM)) (+Ca^2+^ conditions) or binding buffer with no added CaCl_2_ (- Ca^2+^ conditions) by incubation with constant agitation for 30 min/4 °C. Recombinant calcineurin (1 μM) or bovine brain cytosol (∼ 1 mg, dialysed against the appropriate ± Ca^2+^ binding buffer) were then added to immobilised GST proteins in a 100 μl total binding reaction volume and samples incubated with constant agitation for 2 hrs at 4 °C. GST cellulose beads were pelleted by centrifugation (5,000 rpm/1 min/4 °C) and washed with 1 ml of the appropriate binding buffer. This wash step was repeated three times and final bead pellets extracted into 50 μl SDS dissociation buffer (125 mM HEPES pH (6.8), 10% (v/v) sucrose, 10% (v/v) glycerol, 4% (w/v) SDS, 1% β-mercaptoethanol, 2 mM EDTA) and boiling for 5 min. Samples were resolved on SDS-PAGE (12.5% gel) and transferred to nitrocellulose filters for western blotting by transverse electrophoresis. Bound calcineurin was detected using a monoclonal antibody (1:1000, Sigma) followed by incubation with anti-mouse-HRP (1:400, Sigma) and visualisation with ECL reagents. All western blots were quantitated using ImageJ (National Institutes of Health) densitometry software.

### 2.3 Large scale GST protein binding assays

This binding assay is essentially as described in [26]. Briefly, each recombinant GST-fusion protein (5–10 mg), including free GST as control, was immobilised onto 3 ml glutathione-Sepharose 4B resin that had been pre-washed extensively with binding buffer by incubation for 2 h at 4 °C with constant agitation. Clarified bovine brain cytosolic extract was applied to the GST affinity columns and binding allowed to proceed for 16 h at 4 °C with constant agitation. Each column was washed with a minimum 50 volumes of binding buffer and specific Ca^2+^-dependent binding proteins eluted using binding buffer containing no added Ca^2+^(calcium-free or CF buffer). An additional high-salt elution step with binding buffer supplemented with 1 M NaCl and no added Ca^2+^ (High Salt or HS buffer) was used to isolate potential Ca^2+^-independent binding interactions. Eluted protein fractions were concentrated by methanol precipitation and pellets extracted into SDS dissociation buffer by boiling for 5 min. All western blots were quantitated using ImageJ (National Institutes of Health) densitometry software.

### 2.4 Sulfo-SBED cross-linking experiments

The manufacturers protocol provided by Pierce (Rockford, IL, USA) was followed with minor modifications. All steps, except those indicated, were carried out in the dark. Bait recombinant protein of interest (∼5 mg) was dialysed against binding buffer overnight at 4 °C. Immediately before use, the contents of one tube of No-Weigh Sulfo-SBED (Pierce) was dissolved in 22 μl of DMSO, this was then added to 1 ml of dialysed protein. Sulfo-SBED/protein mixes were incubated at room temperature for 30 minutes, centrifuged briefly to remove any precipitated, hydrolysed, Sulfo-SBED, and dialysed overnight against binding buffer. Bovine brain cytosol (500 μl, ∼4 mg protein) dialysed against binding buffer was added to 500μl of the Sulfo-SBED/protein mix. This mixture was incubated on a rotator at room temperature for 1 hour. After this step, samples were exposed to long-wave UV illumination (365 nm) at a distance of ∼5 cm from source for 15 minutes. During this step an appropriate volume of Neutravidin beads (Pierce) were washed extensively in binding buffer, 100 μl added to the bait/cytosol mixture and samples incubated on a rotator for 1 hour at room temperature. Bait/cytosol mixes were then centrifuged at 13,000 rpm for 1 minute, supernatants removed and beads washed with 1 ml of binding buffer. This step was repeated 6 times. Disulfide bond reduction was then achieved by incubating with 1 ml of 50 mM DTT for 1 hour at room temperature. Beads were subsequently centrifuged and washed extensively with binding buffer. After the final wash, 100 μl of SDS dissociation buffer was added to the beads and samples boiled for 10 minutes. Samples were separated on SDS PAGE (12.5% gel) and western blotted with a monoclonal anti-calcineurin antibody (1:1000, Sigma) followed by detection with anti-mouse-HRP (1:400, Sigma) and ECL reagents.

### 2.5 HeLa cell cultures and transfections

HeLa cells were grown in Dulbecco’s modified Eagle’s medium (DMEM, Invitrogen, Paisley, UK) containing 5% foetal bovine serum (Invitrogen), 1% non-essential amino acids (Invitrogen), and 1% penicillin/streptomycin (Invitrogen) at 37 °C in an atmosphere of 5% CO_2_ and maintained at ∼ 1,000,000 cells per 75 cm^2^ flask. 24 hrs prior to transfection, cells were seeded onto glass cover slips on a 24-well plate at ∼ 50,000 cells per well. Transfection mixtures comprised 3 μl Fugene™ (Roche, UK) per 1 μg plasmid DNA in a 100 μl total volume of DMEM and were incubated at room temperature for 30 minutes prior to being added drop-wise to cells. Cells were assayed 24-48 hrs post-transfection. The NFAT-GFP reporter construct was a kind gift from Professor Anjana Rao (Harvard Medical School, Boston, MA, USA).

### 2.6 Ionomycin induced NFAT translocation

Transfected HeLa cells were washed twice in Krebs buffer (NaCl 145 mM; HEPES 20 mM (pH 7.4); D-(+)-Glucose 10 mM; KCl 5 mM; CaCl_2_ 3 mM; MgCl_2_ 1.3 mM; NaH_2_PO_4_ 1.2 mM). Cells were subsequently incubated in 500 μl Krebs containing 3 μM ionomycin or 100 μM histamine for required times before either solubilisation in SDS dissociation buffer, to investigate the phosphorylation state of NFAT via Western blotting, or fixing in 4% formaldehyde to observe the resulting sub-cellular localisation of NFAT post-treatment via confocal microscopy. For western blotting all samples were resolved on SDS-PAGE (12.5% gel) and proteins transferred to nitrocellulose filters by transverse electrophoresis. Filters were probed with monoclonal anti-GFP (1:1000, JL-8 clone, Invitrogen) followed by incubation with anti-mouse-HRP (1:400, Sigma) and protein visualised with ECL reagents.

### 2.7 Confocal microscopy

For confocal laser scanning microscopy, transfected cells were examined with either a Leica TCS-SP-MP microscope or a Leica TCS-SP2-AOBS microscope (Leica Microsystems, Heidelberg, Germany) using a 22 μm pinhole and a 63× water immersion objective with a 1.2 numerical aperture. EGFP was imaged using excitation at 488 nm and light collection at 500-550 nm.

## 3 Results

### 3.1 Direct binding of calcineurin to NCS proteins

**Fig. 1:**
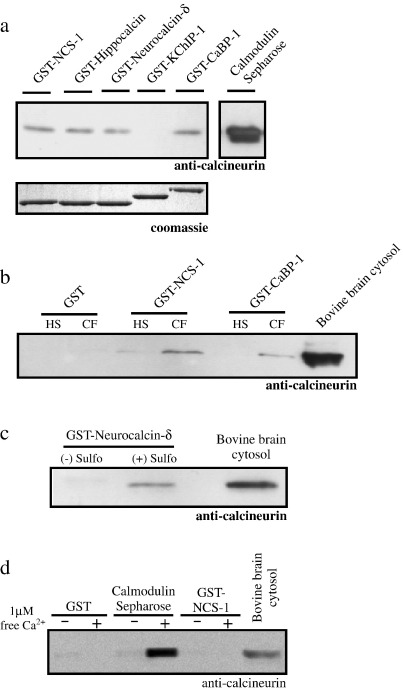
NCS proteins interact with calcineurin in vitro. (a) Upper Panel, Anti-calcineurin western blot of samples from an in vitro binding assay examining interaction of recombinant CN (1 μM) with various GST-tagged NCS proteins (all at 1 μM) and to calmodulin sepharose (1 μM) in the presence of buffer containing 1 μM free Ca^2+^. CN was associated directly with calmodulin sepharose and to a lesser extent with GST-NCS-1, GST-Hippocalcin, GST-Neurocalcin-δ and GST-CaBP1 but not with GST-KChIP1. (a) Lower panel, coomassie blue stained gel highlighting equal loading of GST fusion proteins input into the binding assay. (b) Anti-calcineurin western blot of samples from an in vitro binding assay examining the ability of CN derived from bovine brain cytosolic extract to associate with GST control and GST-NCS proteins in both the absence (High Salt or HS) and presence (Calcium Free or CF) of 1 μM free Ca^2+^. CN was detectable bound to both GST-NCS-1 and GST-CaBP1 only in the presence of free Ca^2+^ and no CN was present in HS samples or GST control protein samples either in the presence or absence of Ca^2+^. The amount of CN in the bovine brain cytosolic extract was shown in a positive control sample of total brain cytosolic lysate resolved on the same gel (Bovine brain cytosol). (c) Anti-calcineurin western blot of samples obtained from a Sulfo-SBED cross-linking experiment (see Materials and Methods for details). CN was detectable in samples from a GST-neurocalcin-δ affinity column treated with the biotin transfer reagent ((+) Sulfo) but not in its absence ((-) Sulfo). Input bovine brain cytosolic extract was resolved on the same gel (Bovine brain cytosol). (d) Anti-calcineurin western blot on samples from in vitro binding experiments involving incubation of bovine brain cytosolic protein extract with GST control protein, calmodulin sepharose and GST-NCS-1 (all at 1 μM) in the absence (-) or presence (+) of 1 μM free Ca^2+^. Total bovine brain cytosolic protein extract was separated on the same gel as a positive control for CN.

A previous in vitro study demonstrated that NCS-1 is capable of stimulating the phosphatase activity of CN but did not examine whether the two proteins directly associate. To confirm a direct interaction we first performed a series of in vitro binding assays utilising various recombinantly expressed and purified NCS proteins to act as baits and where either purified recombinant CN or bovine brain cytosol were employed as a source of prey protein. Initially, purified CN (1 μM) was incubated in the presence of various GST-tagged NCS proteins (also at 1 μM) in binding buffer containing 1 μM free Ca^2+^ (Fig. 1a, upper panel). CaM was included in this assay as a positive control for CN binding (Fig. 1a, upper panel, Calmodulin Sepharose). CN was observed to bind directly to GST-NCS-1, GST-Hippocalcin, GST-Neurocalcin-δ and GST-CaBP1 but not GST-KChIP1, despite equal loading of the proteins (Fig. 1a, lower panel), indicating some degree of interaction specificity. It was also apparent from this analysis that, under identical experimental conditions, seven-fold less CN was bound by GST-tagged NCS proteins than by CaM. We next examined if GST-tagged NCS proteins could efficiently interact with CN present as a component of a complex protein mixture. GST control protein, GST-NCS-1 and GST-CaBP1 (Calcium Binding Protein-1, a calcium sensor more closely related to CaM than the NCS family proteins [28]) were incubated with bovine brain cytosolic protein extract in a large scale pull-down experiment [26] in the presence of 1 μM free Ca^2+^ and Ca^2+^ dependent binding partners eluted by application of Ca^2+^ free buffer containing EGTA and NTA (Fig. 1b, *C*alcium *F*ree (CF) lanes). An additional elution step using 1 M NaCl supplemented buffer was also applied to the samples to elute potential Ca^2+^ independent binding proteins (Fig. 1b, *H*igh *S*alt (HS) lanes). Western blotting for the presence of CN in eluates confirmed a strictly Ca^2+^ dependent interaction with both GST-NCS-1 and GST-CaBP1. No binding was detectable to GST control protein. The signal for CN in the bovine brain cytosolic extract was compared in the same experiment (Fig. 1b, Bovine brain cytosol) and it was clear that CN was not enriched over the brain extract in the Ca^2+^ free eluates.

To provide an independent method to examine the interaction between NCS proteins and CN, we exploited a recently developed bi-functional cross-linking reagent [29] which permits the transfer of a biotin tag from a bait protein of interest to any specifically interacting prey proteins captured following a binding reaction. Samples from a GST-Neurocalcin-δ binding assay prepared via this method were probed with an anti-CN antibody and the presence of the phosphatase confirmed only in samples treated with the cross-linker (Fig. 1c, (+) Sulfo) and no CN was detectable in samples devoid of the reagent (Fig. 1c, (-) Sulfo). Despite use of cross-linking to maintain any low affinity interactions, CN was not enriched in the eluate over brain cytosol input into each condition (Fig. 1c (+) Sulfo vs. Bovine brain cytosol).

The data presented in Fig. 1a suggests that the affinity of CN for CaM may be substantially greater than for the NCS proteins tested. In order to independently verify these observations we further examined the binding of CN from bovine brain cytosol to GST control protein and GST-NCS-1 in direct comparison to CaM (Fig. 1d). We observed Ca^2+^ dependent binding of CN to CaM (Fig. 1d, Calmodulin Sepharose (+)) that was significantly enriched in the eluate compared to bovine brain cytosolic extract, in contrast, at this exposure of the western blot, binding to GST-NCS-1 was barely detectable. On longer exposure to film, NCS-1 bound CN was detectable although it should be noted that results obtained from this small scale binding assay are not directly comparable to data presented in Fig. 1b from a large scale binding assay which employed a greater amount of bovine brain cytosolic prey protein. These data are consistent with a high affinity Ca^2+^ dependent interaction between CaM and CN in contrast to a comparatively weak interaction between NCS-1 and CN.

### 3.2 Overexpression of NCS proteins has no effect on the dephosphorylation of an NFAT-GFP reporter construct in response to cytosolic Ca^2+^ elevation in HeLa cells

Although our in vitro studies pointed to CaM as most likely the predominant interacting/activating partner for CN, we speculated that the situation in the complex physiological setting of mammalian cells might not reflect the results obtained from such a comparatively simple analysis. We therefore attempted to resolve this issue through an examination of a possible interaction between CN and NCS proteins in intact HeLa cells. For these experiments we obtained a GFP tagged NFAT reporter construct [30], encoding the phosphorylated regulatory domain of the phosphatase, that undergoes a detectable dephosphorylation and nuclear translocation in response to sustained increases in [Ca^2+^]i. Our rationale was that should there be a functionally relevant NCS protein/CN interaction in cells then there might be discernable alterations in dephosphorylation kinetics/nuclear translocation of the NFAT reporter in cells [30,31] over-expressing NCS proteins as observed for other NCS-1 specific functions [32-35].

In initial studies, we saw no effect on NFAT dephosphorylation or nuclear translocation of over-expression of wild-type NCS-1, hippocalcin or neurocalcin-δ. Since NCS-1 had been identified in a previous report [25] as an in vitro activator of CN enzymatic activity we decided, however, to focus on this NCS family member for this part of the study. A second reason for examining NCS-1 in further detail also related to the fact that we had at our disposal several mutant NCS-1 constructs one of which has been previously characterised as a dominant inhibitor of NCS-1 function [32]. Such mutants, we reasoned, might assist in unmasking potential functional interactions between NCS-1, CN and NFAT. The first of these mutants, NCS-1^E120Q^, has been employed in a number of studies due to its ability to interact normally with NCS-1 effectors however, as a consequence of impaired Ca^2+^ binding and conformational change, it can then exert *a* dominant inhibitory phenotype [32-36]. The second NCS-1 mutant we employed in this study, NCS-1^G2A^, is myristoylation deficient and, unlike wild type NCS-1 which is constitutively membrane associated through this lipid modification, is entirely cytosolic [37,38]. This protein was included in these studies due to the fact that the majority of cellular CN is similarly cytosolic and hence we hypothesised that the G2A mutation may exaggerate any effects of a potential NCS-1/CN interaction*.*

**Fig. 2:**
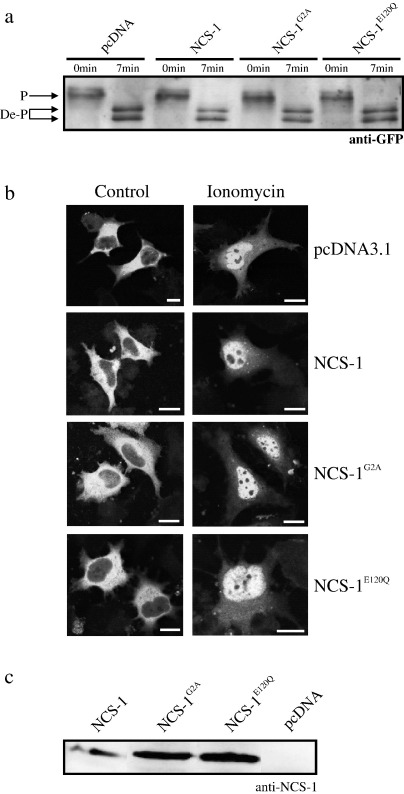
Over-expression of NCS-1 and NCS-1 mutant proteins has no detectable effect on NFAT dephosphorylation or NFAT nuclear translocation. (a) Anti-GFP western blot analysis of samples from HeLa cells co-transfected with an NFAT-GFP reporter construct in addition to control empty vector (pcDNA) or the indicated pcDNA based NCS-1 expression constructs. Cells were treated with Ca^2+^/ionomycin for the indicated times and lysed for SDS-PAGE analysis/western blotting with anti-GFP antibody. Fully phosphorylated NFAT-GFP was detectable in all transfection conditions at the zero time point (P, upper arrow) and dephosphorylation of this reporter was clearly observable after 7 mins of Ca^2+^/ ionomycin treatment with a gel shift to two more rapidly migrating species corresponding to dephosphorylated forms of the protein (DeP, lower arrows). (b) Confocal images of NFAT-GFP fluorescence from HeLa cells transfected and treated as for (a) but subsequently fixed and processed for microscopy. NFAT-GFP fluorescence was diffusely cytosolic and excluded from cell nuclei under control, zero time point, conditions for all transfected constructs tested. On elevation of cytosolic Ca^2+^ for 7 mins (ionomycin) NFAT-GFP fluorescence was observed to translocate from the cytosol to cell nuclei for all transfected constructs analysed, consistent with dephosphorylation of the protein observed in parallel western blot studies (a). Scale bars 10 μm. (c) Western blot of total HeLa cell lysates with an anti-NCS-1 antiserum. Cells transfected identically to those used in functional assays (a and b) were lysed and subjected to western blotting to determine relative over-expression levels of the NCS-1 proteins analysed in this part of the study. Essentially equivalent levels of expression were detected for NCS-1, NCS-1G2A and NCS-1E120Q constructs. No NCS-1 signal was detectable in control empty vector transfected samples (pcDNA).

In an initial analysis, HeLa cells were co-transfected with NFAT-GFP along with control empty vector, NCS-1 or NCS-1 mutant constructs (Fig. 2a). Cells were treated for 7 minutes with ionomycin/Ca^2+^ to elevate [Ca^2+^]_i_, lysed and NFAT-GFP detected by western blotting. A band corresponding to the fully phosphorylated form of NFAT was apparent in 0 time point samples from all transfections (Fig. 2a, P, upper arrow). Dephosphorylation of NFAT to two more rapidly migrating species after sustained [Ca^2+^]_i_ for 7 minutes was observable in all transfected samples and there was no detectable difference in the dephosphorylation pattern obtained between control transfected cells and NCS-1 or NCS-1 mutant transfected conditions (Fig. 2a, DeP, lower arrows). These data are fully consistent with results obtained from parallel confocal imaging analyses of NFAT-GFP nuclear translocation in HeLa cells transfected and treated in an identical manner but which were then fixed for microscopy (Fig. 2b). For all transfection conditions at time 0 of Ca^2+^/ionomycin treatment NFAT-GFP was excluded from cell nuclei and was diffusely cytosolic (Fig. 2b, control, left hand panels), compatible with the presence of the phosphorylated form of the protein (Fig. 2a). After 7 minutes of Ca^2+^/ionomycin treatment NFAT-GFP had redistributed to cell nuclei for all transfection conditions (Fig. 2b, ionomycin, right hand panels) again consistent with the rapid dephosphorylation of the protein (Fig. 2a).

We additionally verified that all NCS-1 expression constructs used in these studies over-expressed in HeLa cells to similar extents when co-expressed with NFAT-GFP (Fig. 2c). Western blotting with an anti-NCS-1 antiserum [39] of total cell lystates from HeLa cells transfected identically to those used in functional assays (Figs. 2a and b) highlighted essentially equivalent levels of over-expression of NCS-1, NCS-1^G2A^ and NCS-1^E120Q^ proteins. There was no detectable NCS-1 protein expression in control, pcDNA transfected, cells (Fig. 2c, pcDNA).

### 3.3 Overexpression of NCS-1, NCS-1^E120Q^ and NCS-1^G2A^ has no effect on the dephosphorylation of an NFAT-GFP reporter construct in response to cytosolic Ca^2+^ elevation in HeLa cells over an acute time course

**Fig. 3:**
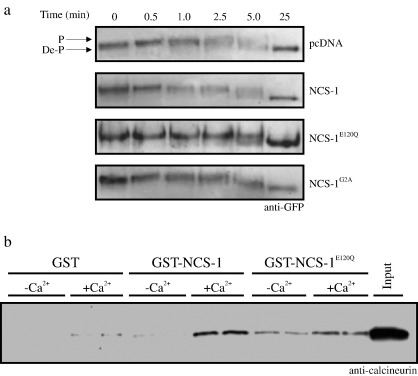
NCS-1 and NCS-1 mutant proteins do not affect dephosphorylation of NFAT-GFP over an acute time course of intracellular Ca^2+^ elevation. (a) Anti-GFP western blots on HeLa cell lysates from cells transfected with NFAT-GFP and the indicated pcDNA based expression constructs. Cells were treated with Ca^2+^/ionomycin for the indicated times and then lysed directly into SDS dissociation buffer, separated on SDS-PAGE (12.5% gel) and transferred to nitrocellulose for western blotting with an anti-GFP antibody. Fully phosphorylated NFAT-GFP (P, upper arrow) present at time 0 for all transfection conditions was dephosphorylated on Ca^2+^/ionomycin treatment as monitored by the appearance of more rapidly migrating species by the 5 min time point for all transfection conditions (De-P, lower arrow). (b) Anti-calcineurin western blot on samples derived from an in vitro binding assay where recombinant CN (1 μM) was incubated with GST control protein, GST-NCS-1 or GST-NCS-1^E120Q^ (all at 1 μM) in the absence (-Ca^2+^) or presence (+Ca^2+^) of 1 μM free Ca^2+^. Samples from duplicate incubations are presented.

Our analyses suggested that, over a 7 minute time course, none of the NCS-1 constructs under examination were capable of affecting the dephosphorylation of NFAT-GFP in response to [Ca^2+^]_i_ elevation and, by inference, nor were they able to influence CN activity in intact mammalian cells. These whole cell data were consistent with our in vitro binding studies however we decided to complete our investigation with two further analyses, firstly examining whether or not NCS-1 might exert a far more subtle effect on the CN/NFAT pathway over an acute time course of [Ca^2+^]_i_ elevation (Fig. 3a). In these experiments, HeLa cells were again transfected with NFAT-GFP along with control or NCS-1 expression constructs however dephosphorylation was monitored over a short 5 minute time course. For all constructs tested NFAT dephosphorylation was clearly apparent by the 5 minute time point (a 25 minute time point was also included to demonstrate complete dephosphorylation of NFAT-GFP) as determined by gel shift of the NFAT-GFP. Analysis of these earlier time points indicated there was no discernable difference in the pattern of dephosphorylation between any of the NCS-1 constructs tested and control transfected cells.

In these experiments the calcium induced elevation in cytosolic free Ca^2+^ was artificially generated by high concentrations of ionomycin and external Ca^2+^. Since NCS-1 has a high affinity for Ca^2+^ (300 nM) one possibility is that it could activate CN under conditions of limited Ca^2+^ elevation thus increasing the overall cellular sensitivity to a Ca^2+^ signal. We completed, therefore, our analyses with an examination as to whether over-expression of NCS-1 or its mutants could influence NFAT dephosphorylation in response to a physiological stimulus. Histamine is a well characterised HeLa cell agonist that generates oscillatory intracellular Ca^2+^ signals [40] and activation of protein kinase C with phorbol esters has been shown to potentiate NFAT driven gene expression elicited by such Ca^2+^ oscillations [41-43]. We stimulated HeLa cells transfected identically to those in Figs. 2 and 3 with 100 μM histamine in both the presence and absence of 50 nM PMA over a three hour time course and assessed the phosphorylation state of NFAT-GFP by western blotting. No dephosphorylation of NFAT-GFP was detectable in these experiments in control vector expressing cells indicating that the stimulus was below the threshold for activation of CN/NFAT-GFP dephosphorylation. Significantly, cells expressing NCS-1, NCS-1^E120Q^ or NCS-1^G2A^ also failed to exhibit dephosphorylation of NFAT-GFP indicating that over-expression of NCS-1 did not enhance the sensitivity of the CN/NFAT signalling pathway to Ca^2+^ signals under these conditions (data not shown)*.*

For NCS-1^E120Q^ to be able to exert a dominant negative effect it would have to bind to CN. In order to validate our use of the NCS-1^E120Q^ mutant as a dominant negative construct in this study we therefore generated a recombinantly purified GST-tagged fusion construct encoding this protein which was used to confirm its ability to bind to purified CN (Fig. 3b). GST-NCS-1E120Q retained almost wild-type CN binding activity in this assay although with less strict Ca^2+^-dependency validating its use in functional assays of NFAT dephosphorylation and nuclear translocation. For GST-NCS-1 we observed a 13.7-fold increase in binding of CN in the presence of Ca^2+^ compared to conditions where Ca^2+^ was absent. In contrast, there was only a 2.2-fold increase in CN binding to GST-NCS-1E120Q in the presence of Ca^2+^.

## 4 Discussion

Diverse Ca^2+^ signals are transduced and decoded within cells by a variety of Ca^2+^-sensing proteins. Of these Ca^2+^ responsive proteins the most intensively studied and best understood is calmodulin (CaM) [2]. CaM is the prototypical member of a super-family of evolutionarily diverse EF-hand containing Ca^2+^ sensors to which the NCS sub-family belongs [23]. Whilst the NCS proteins are only distantly related to CaM, comparative studies have identified significant in vitro overlap in their effector specificities [25]. It was proposed that NCS proteins might represent a set of redundant or alternative modulators of *bone fide* CaM target interactions [25] but the ubiquitous and high level expression of CaM would make this doubtful.

Calcineurin (CN) is a cellular serine/threonine phosphatase critical to the activation of NFAT transcription factors and hence in the control of a variety of fundamental physiological processes [3]. CaM has been identified as the essential co-activator for CN function, however CN has also been identified as a potential target for NCS-1 [25] and as such represents a candidate dual CaM/NCS effector.

A series of possibilities exist as a direct consequence of these observations. Firstly, NCS proteins could indeed simply represent redundant modulators for CaM targets and as such might be expected to elicit responses and to exhibit biochemical properties closely approximating those observed with CaM. Secondly, NCS proteins could feasibly interact with CaM targets in biochemically distinct ways to modulate their activities in specific, CaM independent, manners leading to the overall generation of unique physiological endpoints. The latter has been suggested for dual regulation of voltage-gated Ca^2+^-channels by both CaBP1 and CaM [44]. With specific regard to possible CN/NFAT activation by NCS-1, this possibility is intriguing in light of data identifying NFAT dependent gene expression as important to aspects of neuronal cell function. Thirdly, the possibility also exists that interactions observed between NCS proteins and CaM targets are physiologically irrelevant in cells. In this study we have attempted to resolve these issues through an investigation of firstly the biochemical characteristics of NCS protein interactions with CN, and secondly by examining the potential consequences of NCS protein function on activation of the classical CaM-CN-NFAT pathway in mammalian cells.

In a series of in vitro biochemical assays we were able to show that there exists a selective and Ca^2+^ dependent interaction between various NCS proteins and both purified recombinant CN, and CN derived from bovine brain cytosolic protein extract. We noted during these analyses however that significantly more CN bound to immobilized CaM. These data are consistent with the findings of Schaad *et al.* indicating that NCS-1 is able to stimulate the phosphatase activity of CN in vitro but to a lesser extent than observed for CaM activation. In experiments utilising bovine brain cytosol as a source of prey protein the difference in binding of the NCS proteins for CN in comparison to CaM was more greatly magnified, and whilst considerable quantities of CN could be affinity purified by a calmodulin sepharose column, the amount of CN associated with NCS family members under identical experimental conditions was considerably lower. Previous work using a biotinylated protein overlay assay also found much less binding of CN to NCS-1 compared to CaM [45]. These data led us to conclude that the CaM/CN interaction was likely to be of a higher affinity than for interactions between CN and NCS proteins. Indeed, published biochemical parameters suggest that the in vitro *K_d_* for CaM/CN interaction may be ≤ 0.1 nM [46] with that reported for NCS-1/CN being 350 nM [25]. The in vitro affinity of CaM for CN is therefore three orders of magnitude greater than that determined for NCS-1 which would agree with the binding data presented in this study. Importantly, CaM is expressed at 5-200 fold higher concentrations in cells than NCS-1 [25] and so CN would be predicted to be bound to CaM rather than NCS-1 at elevated [Ca^2+^]_i_ levels.

To assess whether NCS proteins were capable of modulating CN activity in a cellular environment we employed the dephosphorylation and nuclear translocation of a characterised NFAT-GFP reporter construct as a probe of CN phosphatase activity. Co-expression of NCS-1 did not alter the basal phosphorylation state of NFAT-GFP compared with control transfected cells nor did it exert a detectable effect on NFAT-GFP dephosphorylation/nuclear translocation after sustained elevation of [Ca^2+^]_i_ during single endpoint and acute time course analyses. Co-expression of the NCS proteins Hippocalcin, Neurocalcin-δ [26], and the more closely related CaBP1 (which shares 56% similarity with CaM [47]) were similarly without effect in analyses of NFAT-GFP dephosphorylation (data not shown). From these analyses we cannot formally exclude the possibility that NCS family proteins may activate a pool of cellular CN that does not signal via NFAT dephosphorylation. This seems unlikely however taking into account our biochemical data and the fact that in numerous other studies assessing diverse triggers of CN activity, concomitant activation of the NFAT pathway is always detected.

In an extension to these studies we also examined the effects of two NCS-1 mutant proteins, NCS-1^E120Q^ and NCS-1^G2A^, on NFAT-GFP dephosphorylation in HeLa cells. We were able to demonstrate that the E120Q mutant of NCS-1 [32] retained approximately wild type binding properties with respect to interaction with purified CN. However, this protein, which has been routinely observed to exert a dominant negative phenotype in various other studies of NCS-1 function [32-35], was unable to generate detectable alterations in NFAT-GFP dephosphorylation. In an effort to try and understand if NCS-1 was unable to mediate an in vivo modulation of CN activity due to its restricted membrane localisation we also examined NFAT-GFP phosphorylation in the presence of co-expressed NCS-1^G2A^, a protein that fails to become myristoylated and as a result remains cytosolic. Since the bulk of cellular CN is also cytosolic we reasoned that expression of the G2A mutant might bias any interaction with CN to generate an observable alteration in dephosphorylation of NFAT-GFP. Our analysis of this mutant indicated that it elicited no significant change in the kinetics of NFAT-GFP dephosphorylation compared to control transfected cells. In related experiments we tested the ability of NCS-1 and NCS-1 dominant inhibitory mutants to influence NFAT dephosphorylation in response to physiological stimuli. We observed no detectable changes in NFAT dephosphorylation in the presence of over-expressed NCS-1 proteins suggesting that in addition to sustained elevation of cytosolic free Ca^2+^, NCS-1 proteins are similarly unable to stimulate NFAT dephosphorylation in response to agonist derived Ca^2+^ signals that were below the threshold for significant activation of CN and NFAT dephosphorylation.

Collectively our findings would argue that, although NCS proteins are capable of interacting with CN in vitro, they may be unable to act as functionally relevant activators of the enzyme in a cellular context. The possibility remains that there may be some level of interplay between NCS proteins and CaM within protein complexes, exemplified by observations that both CaM and CaBP1 interact with IP3-receptors [40,48,49], that both NCS-1 and CaM are able to interact with Ca^2+^ channels [50] and G-protein coupled receptor kinases [34,51,52] and that CaM, NCS-1 and CaBP1 have all been shown to modulate the activity of the transient receptor potential channel, TRPC5 [35]. It is impossible to formally rule out direct modulation of CN by NCS proteins under all physiological conditions based on the data presented in this study however we have provided data to challenge the idea that CN is a cellular target of the NCS protein family and instead that CaM appears the more likely regulator of this key signalling phosphatase.
